# Surgical Outcomes of in situ Internal Thoracic Artery Grafting in Patients with Ipsilateral Upper-Extremity Arteriovenous Fistulas

**DOI:** 10.1093/icvts/ivag231

**Published:** 2026-08-26

**Authors:** Chijioke Chukwudi, Ruby Singh, Arian Mansur, Benjamin Grobman, Patrick Udeh, Jordan Bloom, George Tolis, David A D’Alessandro, Thoralf M Sundt, Asishana A Osho

**Affiliations:** Department of Cardiothoracic Surgery, University of Pittsburgh Medical Center, Pittsburgh, PA 15213, United States; Division of Cardiac Surgery, Mass General Brigham, Boston, MA 02114, United States; Department of Cardiothoracic Surgery, Stanford Medicine, Palo Alto, CA 94305, United States; Harvard Medical School, Boston, MA 02114, United States; Division of Cardiac Surgery, Mass General Brigham, Boston, MA 02114, United States; Division of Cardiac Surgery, Mass General Brigham, Boston, MA 02114, United States; Division of Cardiac Surgery, Mass General Brigham, Boston, MA 02114, United States; Division of Cardiac Surgery, Mass General Brigham, Boston, MA 02114, United States; Division of Cardiac Surgery, Mass General Brigham, Boston, MA 02114, United States; Division of Cardiac Surgery, Mass General Brigham, Boston, MA 02114, United States

**Keywords:** coronary artery bypass grafting, internal thoracic artery, haemodialysis, arteriovenous fistulas

## Abstract

**Objectives:**

The use of in situ internal thoracic artery (ITA) grafts ipsilateral to upper extremity arterio-venous fistulas (UE-AVFs) during coronary artery bypass grafting (CABG) in dialysis-dependent patients raises concerns of coronary steal and compromised graft perfusion. However, the clinical significance of ITA-AVF laterality remains uncertain.

**Methods:**

We retrospectively analysed dialysis-dependent patients who underwent CABG between February 2015 and December 2023 at 2 institutions. Patients with non-UE-AVF access, total vein grafting, or composite vein-artery configurations were excluded. The final cohort (*N *= 122) was divided into 2 groups: ipsilateral in situ ITA grafts (*N* = 66) and contralateral in situ or free ITA grafts (*N* = 56). The primary outcome was the incidence of major adverse cardiovascular and cerebrovascular events (MACCE); secondary outcomes included perioperative complications, haemodialysis-induced ischaemia, and 3-year survival.

**Results:**

Baseline and operative characteristics were largely comparable between groups. Perioperative outcomes, including surgical site infection, 30-day mortality, and prolonged ventilation, showed no significant differences. Over a median follow-up of 2.4 years, MACCE occurred in 44% of patients (39% ipsilateral vs 50% contralateral/free ITA; *P* = .24), with no significant differences in 3-year mortality (29% vs 21%; *P* = .352). Multivariable analyses showed no association between ITA-AVF laterality and MACCE (adjusted odds ratio 0.68, *P* = .31) or 3-year mortality (adjusted hazard ratio 1.56, *P* = .291).

**Conclusions:**

In this modestly sized retrospective cohort, ipsilateral in situ ITA grafting was not associated with significantly worse perioperative or long-term outcomes. Our findings should be interpreted as hypothesis-generating, and larger multicentre studies are needed to more definitively evaluate the safety of this grafting strategy in dialysis-dependent patients with UE-AVFs.

## Graphical abstract

**Figure:**
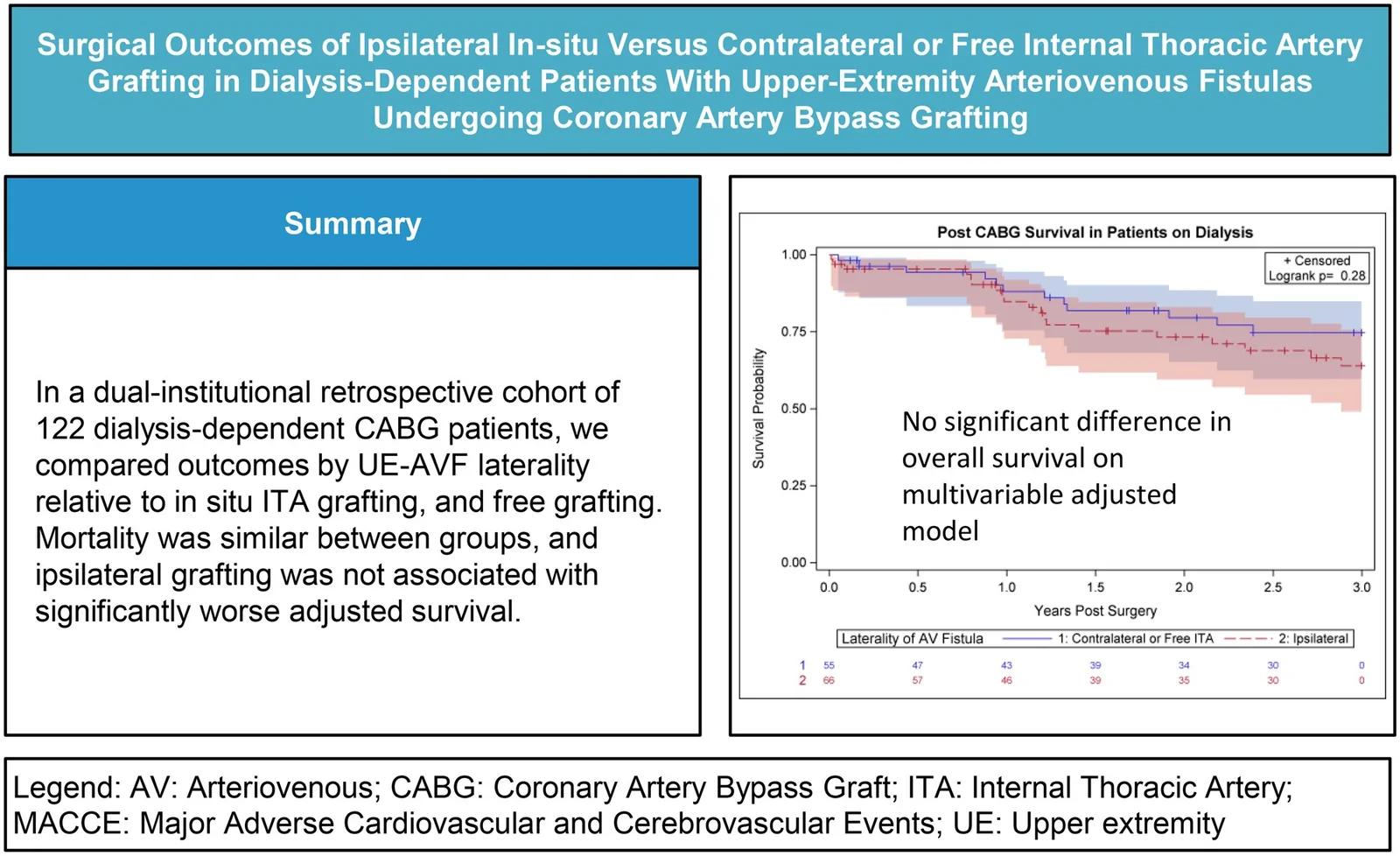

## INTRODUCTION

Multi-vessel coronary artery disease (CAD) is highly prevalent in patients with end-stage renal disease (ESRD) and is associated with substantially increased morbidity and mortality.^1^ Compared to the general population, ESRD patients undergoing coronary artery bypass grafting (CABG) have a significantly worse prognosis with higher rates of perioperative mortality and diminished long-term survival.^2–4^ Although these outcomes may be attributable to the deleterious effects of chronic uraemia, and the multiple comorbidities harboured by this patient population, the choice of vascular conduit and revascularization strategy during CABG may play a significant role in the observed outcomes.

The internal thoracic artery (ITA) is the choice vascular conduit for coronary revascularization, and in situ ITA grafting, particularly to the left anterior descending (LAD) artery, has been demonstrably shown to maintain long-term patency while enhancing survival and freedom from major adverse cardiovascular and cerebrovascular events (MACCE).^5^^,^^6^ However, in ESRD-patients dialyzed through an upper extremity AV-fistula (UE-AVF), concerns exist that utilization of ipsilateral in situ ITA grafts may result in dialysis-associated haemodynamic alteration and under-perfusion of the distal target.^7^ This may manifest acutely during haemodialysis sessions as angina, or chronically as progressively diminishing myocardial function and increased risk of adverse cardiovascular events.

It remains unclear whether utilization of ipsilateral in situ ITA grafts engenders worse outcomes, and whether contralateral in situ ITAs or free grafts may provide superior results. Our study aims to address a critical gap in the literature by evaluating clinical outcomes associated with ipsilateral in situ ITA grafting. Specifically, we compare ipsilateral in situ ITA use with contralateral and free graft configurations to determine whether AVF laterality influences survival, rates of haemodialysis-induced angina, or MACCE. By leveraging a cohort with extended follow-up, our aim is to generate evidence that can guide revascularization strategy and improve CABG outcomes in this high-risk population.

## PATIENTS AND METHODS

### Study population

**Figure 1.**
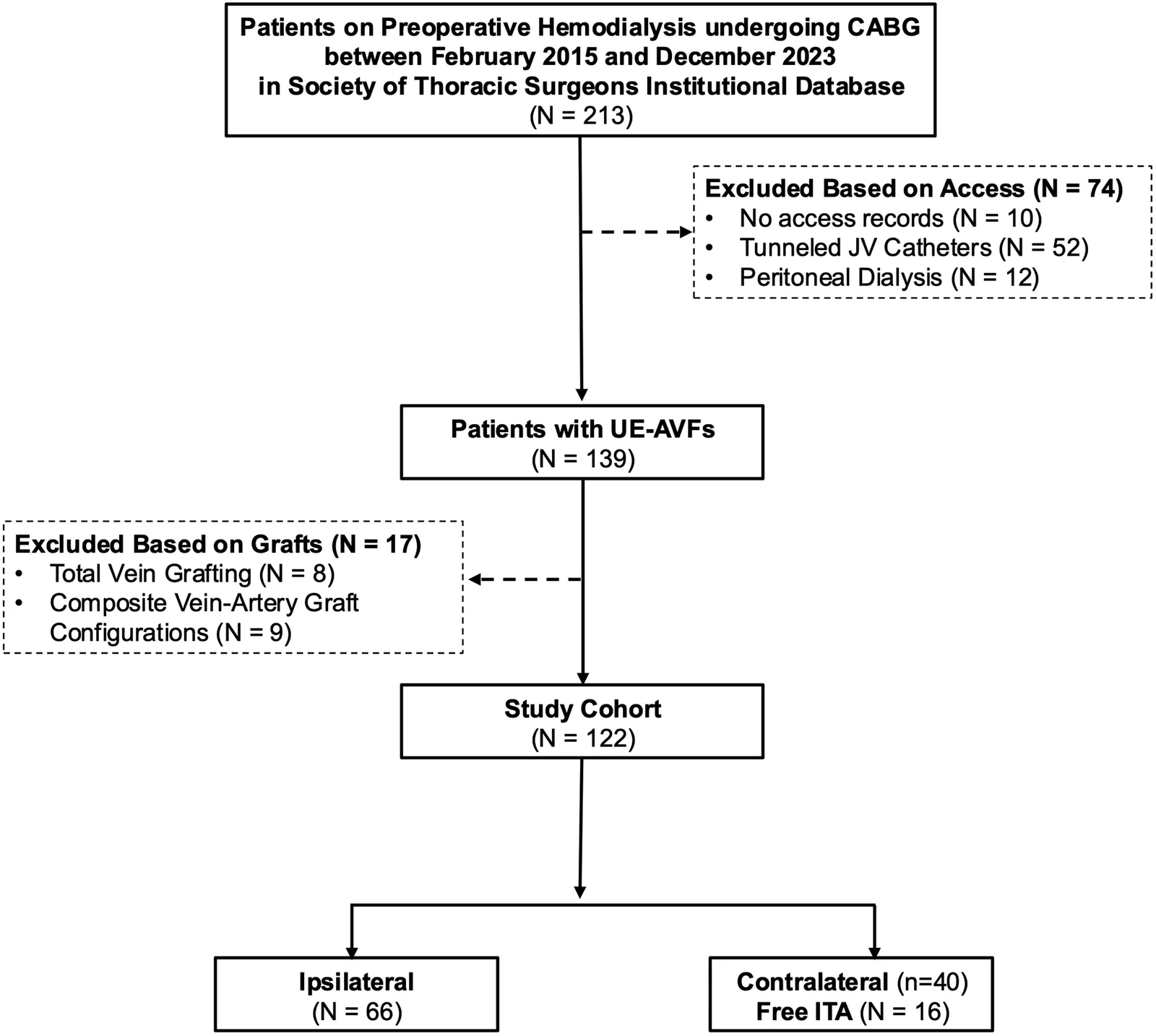
Cohort Selection Flowchart. CABG patients on dialysis (*N* = 213) were screened; 122 met criteria and were grouped by ITA graft type: ipsilateral in situ (*N* = 66) vs. contralateral/free ITA (*N* = 56). Abbreviations: CABG, coronary artery bypass grafting; ITA, internal thoracic artery; UE-AVF, upper extremity arterio-venous fistula

Institutional Society of Thoracic Surgeons datasets were queried to identify dialysis-dependent ESRD patients who underwent CABG between February 2015 and December 2023 at 2 institutions. A total of 213 patients were identified. We excluded patients for whom dialysis access was obtained from an alternate route than an upper extremity AV-fistula (UE-AVF) (tunnelled jugular venous catheter [52; 24%], peritoneal dialysis [12; 6%]) and those for whom access records were unavailable (10; 5%) (**Figure 1**). Next, we excluded patients who received solely vein grafts (8; 4%) and those with composite graft configurations (vein-arterial or arterial Y/T configurations) (9; 4%). Thus, 122 patients met our inclusion criteria and were split into 2 groups. The first group consisted of patients who received an in situ ITA graft ipsilateral to their UE-AVF (66; 54%), while the second group was a composite of patients who either received an in situ ITA graft contralateral to their UE-AVF or a free ITA graft anastomosed to the diseased vessel and aorta (56; 46%). Contralateral in situ and free ITA grafts were combined because both configurations avoid reliance on an in situ ITA graft arising from the subclavian artery ipsilateral to the UE-AVF, which was the primary exposure of interest. Median follow-up duration was 2.4 years (interquartile range [IQR]: 1-3.9).

### Operative method

Operative strategy was determined by the attending surgeon based on patient anatomy, coronary targets, conduit availability, aortic quality, and overall operative risk. Most cases were performed using conventional on-pump CABG with cardioplegic arrest. After median sternotomy, the ITA was harvested as an in situ or free conduit according to the planned revascularization strategy, and additional venous or arterial conduits were used at the surgeon’s discretion. Following systemic heparinization, cardiopulmonary bypass (CPB) was established and myocardial protection was achieved using institutional standard techniques. In patients with severe ascending aortic calcification or other concerns regarding aortic manipulation, cross-clamping and proximal aortic anastomoses were avoided or modified at the discretion of the operating surgeon. Intraoperative assessment of conduit quality and graft flow was performed selectively based on surgeon preference and clinical context.

### Data source

Data on patient baseline characteristics, operative characteristics, and early postoperative outcomes were obtained from our institutional STS database. The laterality of the AV fistula, adverse events, and long-term outcomes were assessed by chart review.

### Ethics statement

This retrospective study used stored clinical data only and did not involve collection or storage of biological material. Data use and storage were approved by the Mass General Brigham IRB (Protocol# 2004P001528) and were conducted in accordance with applicable data protection requirements and the WMA Declaration of Taipei.

### Outcomes

The primary outcome was the postoperative occurrence of MACCE. We defined MACCE as cardiac death, myocardial infarction, new decompensated congestive heart failure (CHF), cardiac arrest, repeat revascularization, or stroke. Cardiac death was defined as death from lethal arrhythmia, acute coronary syndrome, decompensated heart failure, or cardiogenic shock.

Secondary outcomes included short-term outcomes of surgical site infection, re-bleeding, sepsis, prolonged ventilation, index hospital length of stay, ICU length of stay as well as long-term outcomes of haemodialysis-induced ischaemia, overall time all-cause mortality, 3-year mortality, and 3-year survival conditional on 1-year survival.

### Statistical analysis

Baseline demographic and clinical characteristics and immediate postoperative outcomes were examined by AV-fistula laterality. Continuous parametric variables are presented as mean (standard deviation), assessed using 2-sample *t*-tests, and non-parametric variables as median (IQRs), assessed using Wilcoxon’s Rank Sum test. Normality was visually assessed using histograms. Categorical variables are presented as frequency (percentages) and assessed using the Chi-squared test or Fisher’s exact test when expected cell counts were less than 5.

Major adverse cardiovascular and cerebrovascular events were examined using univariable and multivariable logistic regression. Three-year mortality was assessed using univariable and multivariable Cox regression. Variable selection for multivariable models was based on *P* values of < .1 on univariable analyses and clinical significance.

Less than 5% of data are missing in variables used in the analysis, and a complete case analysis was performed. A 2-sided *P* value of less than .05 was considered statistically significant. All analyses were performed using SAS Enterprise Guide 8.3 (SAS Institute, Cary, NC, United States).

## RESULTS

### Patient baseline characteristics

The median age of the cohort was 63 years (IQR: 57-71), with no significant difference between groups (*P* = .227) (**Table 1**). Body mass index (BMI) was also comparable across both groups (30.8 vs 27.7; *P* = .059). Female sex distribution was similar between groups (21% vs 25%; *P* = .62), and there were no significant differences in racial distribution (*P* = .706). Comorbidities such as hypertension (*P* = .179), diabetes (*P* = .174), atrial fibrillation (*P* = .204), peripheral vascular disease (*P* = .129), CHF (*P* = .905), stroke (*P* = .918), hyperlipidaemia (*P* = .764), acute coronary syndrome (*P* = .774), prior Percutaneous Coronary Intervention (PCI) (*P* = .915), obstructive sleep apnoea (*P* = .115), and liver disease (*P* = .725) were similar between groups. Chronic lung disease was significantly more common in the ipsilateral group (23% vs 5%; *P* = .006). Preoperative creatinine levels (median 5.7 mg/dl in both groups; *P* = .768) and left ventricular ejection fraction (LVEF; *P* = .174) were comparable. Smoking history was similar between groups (53% vs 46%; *P* = .467). The number of diseased vessels was not significantly different (*P* = .452), with 3-vessel disease being the most common in both groups. Overall, 40 patients (33%) had a right-sided AV-fistula compared to 82 (67%) with a left AV-fistula. Ipsilateral grafts were more common in patients with a left-sided AVF (97% vs 3%), whereas contralateral/free grafts were more common in patients with right-sided AVFs (68% vs 32%). Overall rates of subclavian stenosis were similar across groups (12% vs 20%; *P* = .254). Amongst the 18 patients with left-sided AVFs who received contralateral/free grafts, only 1 had subclavian stenosis, which was detected postoperatively, suggesting that concern for coronary steal, rather than documented subclavian stenosis, was the primary determinant in choosing a contralateral or free ITA graft for these patients.

**Table 1.** Baseline Characteristics

	Overall (*n* = 122)	Ipsilateral (*n* = 66)	Contralateral +free ITA (*n* = 56)	*P* value	Missing
Age	63 (57-71)	64 (58-72)	61 (56-71)	.227	0
BMI	28.9 (24.7-32.8)	30.8 (25.2-34.8)	27.7 (24.5-31.5)	.059	0
Female sex	28 (23%)	14 (21%)	14 (25%)	.62	0
Race				.706	6
White	86 (74%)	49 (78%)	37 (70%)		
Black	11 (10%)	5 (8%)	6 (11%)		
Asian	6 (5%)	2 (3%)	4 (8%)		
Hispanic	13 (11%)	7 (11%)	6 (11%)		
Hypertension	117 (96%)	65 (99%)	52 (93%)	.179	0
Diabetes	96 (79%)	55 (83%)	41 (73%)	.174	0
Atrial fibrillation	21 (17%)	14 (21%)	7 (13%)	.204	0
Creatinine	5.7 (4.6-7.4)	5.7 (4.8-7.4)	5.7 (4.4-7.7)	.768	0
Peripheral vascular disease	39 (32%)	25 (38%)	14 (25%)	.129	0
Chronic lung disease	18 (15%)	15 (23%)	3 (5%)	**.006**	1
History of smoking	61 (50%)	35 (53%)	26 (46%)	.467	0
Congestive heart failure	72 (60%)	39 (60%)	33 (59%)	.905	1
LVEF	54 (42.5-64)	55 (45-65)	50 (40-62)	.174	2
Stroke	17 (14%)	9 (14%)	8 (14%)	.918	0
Hyperlipidaemia	110 (90%)	60 (91%)	50 (89%)	.764	0
Acute coronary syndrome	68 (56%)	36 (55%)	32 (57%)	.774	0
Prior PCI	42 (34%)	23 (35%)	19 (34%)	.915	0
Obstructive sleep apnoea	37 (30%)	24 (36%)	13 (23%)	.115	0
Liver disease	8 (7%)	5 (8%)	3 (5%)	.725	0
Number of diseased vessels				.452	0
1	12 (10%)	6 (9%)	6 (11%)		
2	29 (24%)	13 (20%)	16 (29%)		
3 or more	81 (66%)	47 (71%)	34 (61%)		
Side of arteriovenous fistula				**<.0001**	0
Right	40 (33%)	2 (3%)	38 (68%)		
Left	82 (67%)	64 (97%)	18 (32%)		
Subclavian stenosis	19 (16%)	8 (12%)	11 (20%)	.254	0

Abbreviations: BMI, body mass index; ITA, internal thoracic artery; LVEF, left ventricular ejection fraction.

### Operative characteristics

Rates of redo sternotomy were low in both groups (5% in the ipsilateral group vs 2% in the contralateral/free ITA group; *P* = .623) (**Table 2**). Elective procedures accounted for 35% of the overall cohort, with no significant difference between groups (33% vs 36%; *P* = .703). Median CPB times were 98 min (IQR: 79-148) for the ipsilateral group and 118 min (IQR: 78-167) for the contralateral/free ITA group (*P* = .387). Cross-clamp times were also similar between groups (76 vs 85 min; *P* = .234).

**Table 2.** Operative Characteristics

	Overall (*n* = 122)	Ipsilateral (*n* = 66)	Contralateral +free ITA (*n* = 56)	*P* value	Missing
Redo sternotomy	4 (3%)	3 (5%)	1 (2%)	.623	0
Elective procedure	41 (35%)	21 (33%)	20 (36%)	.703	0
CPB time	105 (78.5-154.5)	98 (79-148)	118 (78-167)	.387	2
Cross-clamp time	78 (61-118)	76 (61.5-108)	85 (58-135)	.234	3
Concomitant operations				.875	0
None	82 (67%)	47 (71%)	35 (63%)		
MV repair/replacement	10 (8%)	5 (8%)	5 (9%)		
AV repair/replacement	12 (10%)	6 (9%)	6 (11%)		
Other	13 (11%)	6 (9%)	7 (13%)		
>1 concomitant procedure	5 (4%)	2 (3%)	3 (5%)		
LIMA use				.725	4
To LAD	115 (98%)	64 (98%)	51 (96%)		
To LCX	1 (1%)	0 (0%)	1 (2%)		
To OM	2 (2%)	1 (2%)	1 (2%)		
To RCA	0 (0%)	0 (0%)	0 (0%)		
RIMA use				1	
To LAD	2 (20%)	1 (20%)	1 (20%)		
To Diag	1 (10%)	1 (20%)	0 (0%)		
To OM	6 (60%)	3 (60%)	3 (60%)		
To PDA	1 (10%)	0 (0%)	1 (20%)		
SVG use					
To OM	74 (71%)	37 (65%)	37 (77%)	.173	17
To Diag	24 (23%)	16 (28%)	8 (17%)	.166	17
Bilateral ITA use	7 (6%)	4 (6%)	3 (5%)	1	0
All arterial conduits	16 (13%)	9 (14%)	7 (13%)	.853	0

Abbreviations: AV, arterio-venous; CPB, cardiopulmonary bypass; ITA, internal thoracic artery; LAD, left anterior descending; LIMA, left internal mammary artery; LCX, Left Circumflex; PDA, Posterior Descending Artery; RCA, Right Coronary Artery; MV, mitral valve; OM, obtuse marginal; RIMA, right internal mammary artery; SVG, saphenous vein graft.

Concomitant procedures were performed at comparable rates across groups (*P* = .875), with 67% undergoing isolated CABG. Mitral valve (MV) repair/replacement, aortic valve (AV) repair/replacement, and other procedures were distributed similarly between the 2 cohorts. Use of the left internal mammary artery (LIMA) to the LAD was nearly universal in both groups (98% vs 96%; *P* = .725). Right internal mammary artery (RIMA) usage patterns were consistent across groups, most commonly to the obtuse marginal (OM) artery.

Saphenous vein graft (SVG) use was common, with SVGs to the OM used in 71% of patients (65% ipsilateral vs 77% contralateral/free ITA; *P* = .173), and SVGs to the diagonal artery used in 23% of patients (28% vs 17%; *P* = .166). Bilateral ITA use was rare (6% overall; *P* = 1), as was exclusive use of arterial conduits (13% overall; *P* = .853).

### Perioperative outcomes

Surgical site infections occurred in 5% of patients overall, with similar rates between the ipsilateral (3%) and contralateral/free ITA (7%) groups (*P* = .412) (**Table 3**). Thirty-day mortality was 4% for the overall cohort, with no significant difference between groups (6% vs 2%; *P* = .373). Postoperative rebleeding and sepsis were rare (2% and 1%, respectively) and evenly distributed across groups (both *P* ≥ 0.459). Rates of prolonged ventilation were comparable (18% in the ipsilateral group vs 13% in the contralateral/free ITA group; *P* = .458). Median hospital length of stay was 10 days (IQR: 7-14 overall), and ICU stay was 75 hours (IQR: 46-115), with no significant differences between groups (*P* = .547 and *P* = .407, respectively). These findings suggest comparable short-term outcomes regardless of AVF laterality.

**Table 3.** Perioperative Outcomes

	Overall (*n* = 122)	Ipsilateral (*n* = 66)	Contralateral + free ITA (*n* = 56)	*P* value
Surgical site infection	6 (5%)	2 (3%)	4 (7%)	.412
30-Day mortality	5 (4%)	4 (6%)	1 (2%)	.373
Postoperative rebleeding	2 (2%)	1 (2%)	1 (2%)	1
Sepsis	1 (1%)	0 (0%)	1 (2%)	.459
Prolonged ventilation	19 (16%)	12 (18%)	7 (13%)	.458
Hospital length of stay, days	10 (7-14)	9 (7-14)	10 (7-15)	.547
ICU length of stay, hours	75 (46-115)	73 (45-98)	87 (47-137)	.407

Abbreviation: ITA, internal thoracic artery.

### Long-term outcomes

Long-term outcomes were not statistically different between the ipsilateral and contralateral/free ITA groups (**Table 4**). Haemodialysis-induced ischaemia was rare, occurring in only 1 patient in each group.

**Table 4.** Long-Term Outcomes

	Overall (*n *= 122)	Ipsilateral (*n* = 66)	Contralateral + free ITA (*n* = 56)	*P* value
HD-induced ischaemia	2 (2%)	1 (2%)	1 (2%)	1
Major adverse cardiovascular event	54 (44%)	26 (39%)	28 (50%)	.24
All time mortality	46 (38%)	27 (41%)	19 (34%)	.428
3-Year mortality	31 (25%)	19 (29%)	12 (21%)	.352
3-Year mortality conditional on 1 year survival	16 (15%)	10 (18%)	6 (12%)	.422

Abbreviation: ITA, internal thoracic artery; HD, Hemodialysis.

The incidence of MACCE was 44% overall, with a non-significant trend towards lower rates in the ipsilateral ITA group (39% vs 50%; *P* = .24). Univariable and multivariable logistic regression analysis revealed no significant association between ITA graft-AV fistula laterality and MACCE (**Tables S1 and S3**). Despite the statistical non-significance, the univariable model demonstrated a 46% lower odds of MACCE (odds ratio [OR] of 0.54; 95% CI: 0.25-1.18; *P* = .124]), and the multivariable model demonstrated 32% lower odds (adjusted odds ratio [aOR]: 0.68; 95% CI: 0.32-1.44; *P* = .31) in patients with ipsilateral AV fistula compared to those with contralateral + free ITA when adjusted for age, diabetes, hypertension, smoking history, and peripheral vascular disease.

**Figure 2.**
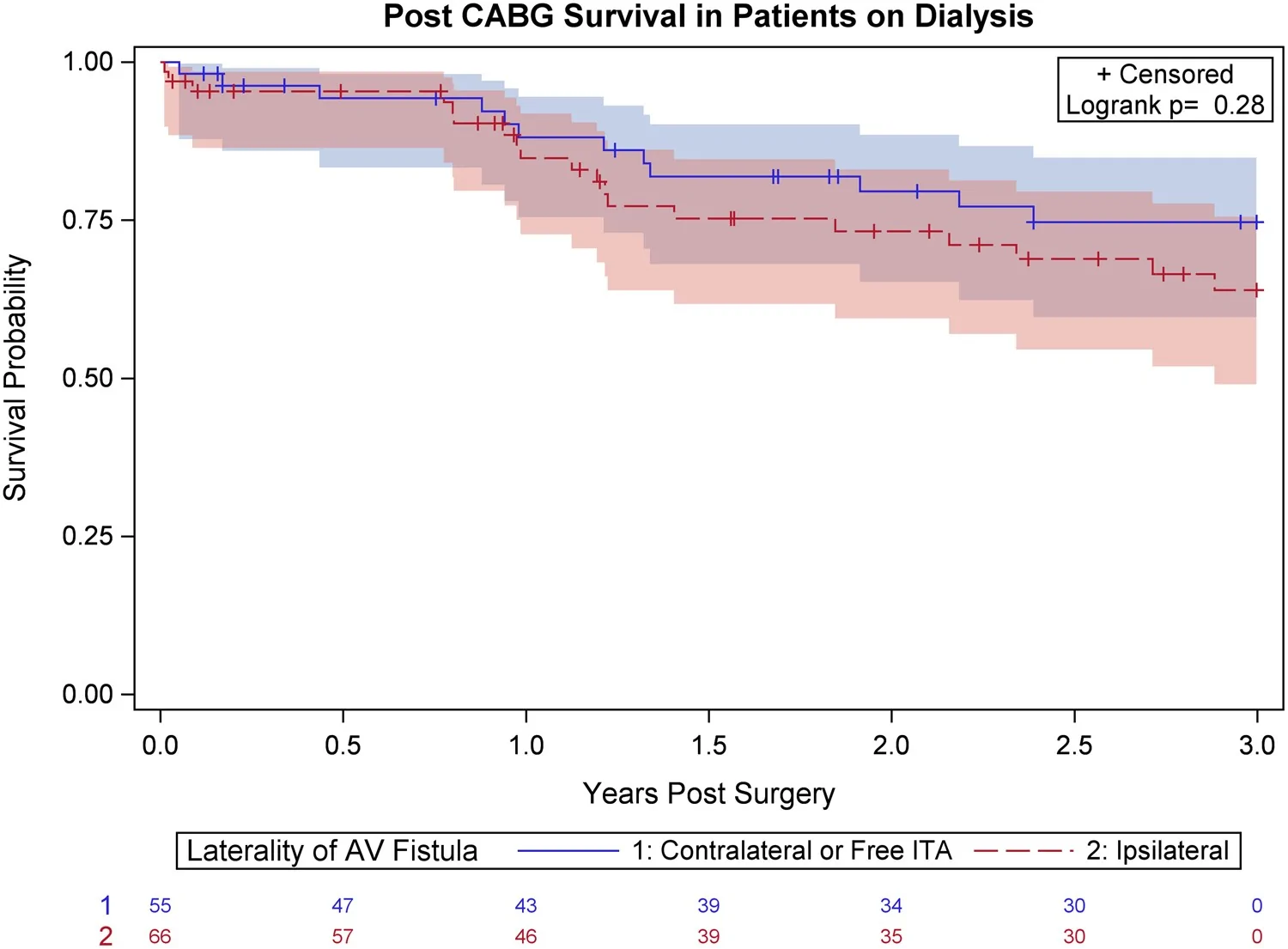
Kaplan-Meier Survival by ITA-AVF Laterality. Three-year survival estimates for CABG patients on dialysis, stratified by ITA graft laterality. Group 1 (blue): contralateral in situ or free ITA; group 2 (red dashed): ipsilateral in situ ITA. Shaded areas show 95% CI. No significant difference (log-rank *P* = .28); tick marks denote censoring. Abbreviations: AV, arterio-venous; CABG, coronary artery bypass grafting; ITA, internal thoracic artery

All-time mortality was 41% in the ipsilateral group vs 34% in the contralateral + free ITA group (*P* = .428). Three-year mortality was 29% in the ipsilateral group vs 21% in the contralateral + free ITA group (*P* = .352) (**Figure 2**), and among those who survived the first year, conditional 3-year mortality remained statistically similar (18% vs 12%; *P* = .422) (**Figure S1**).

On univariable Cox regression analysis, there was no statistically significant difference in the risk of 3-year mortality in the ipsilateral AV fistula group compared to group contralateral + free ITA group. However, the risk of 3-year mortality was 49% higher compared to the contralateral + free ITA group (HR = 1.49; 95% CI: 0.72-3.07, *P* = .281; **Table S4**). Similarly, multivariable Cox regression analysis also showed no significant association between ITA graft—AV fistula laterality and 3-year mortality (**Table S4**). But the adjusted hazard ratio (aHR) for patients with ipsilateral AV fistula showed a 56% higher risk of mortality (aHR = 1.56; 95% CI: 0.68-3.58; *P* = .291) as compared to the contralateral + free ITA group when adjusted for the patient’s history of peripheral vascular disease and pre-operative LVEF.

In an unadjusted sensitivity analysis limited to patients undergoing isolated CABG, rates of haemodialysis-induced ischaemia remained low and similar between groups, occurring in 1 patient in the ipsilateral group and 1 patient in the contralateral/free ITA group. Three-year mortality was also similar between groups (23% vs 26%; *P* = .81). Major adverse cardiovascular and cerebrovascular events occurred less frequently among patients who received ipsilateral in situ ITA grafts compared with those who received contralateral/free ITA grafts (34% vs 57%; *P* = .037) (**Table S5**).

### Potential impact of subclavian stenosis in patients with ipsilateral grafts

Preoperative imaging was available for most patients, and surgeons largely avoided grafting with ITAs from stenosed subclavian arteries. Most diagnoses of subclavian stenosis in the ipsilateral group were made postoperatively. Subclavian artery stenosis (arterial occlusion of 50% or greater) was identified in 8 patients (12%) who received ipsilateral ITA grafts, and in 11 patients (20%) who underwent contralateral or free ITA grafting. Interestingly, among those with subclavian stenosis who received ipsilateral grafts, the all-time mortality rate was lower than that of the overall ipsilateral cohort (25% vs 41%). However, this subgroup experienced a markedly higher incidence of MACCE (62.5% vs 39%).

## COMMENT

Our study found no significant difference in rates of perioperative complications, early or late mortality, and MACCE between patients regardless of ITA-AVF laterality. These findings suggest that using in situ ITA grafts ipsilateral to UE-AVFs may be a safe and appropriate revascularization strategy for dialysis-dependent patients.

Despite early reports suggesting that UE-AVFs may cause coronary steal when paired with ipsilateral ITAs, the evidence and clinical significance remain unclear. Isolated case studies^7–10^ have described reduced flow in ipsilateral ITAs during dialysis and angiographic signs of retrograde flow. Yet, observational studies have largely failed to demonstrate an association between ITA-AVF laterality and adverse outcomes.^11–14^ Takami et al,^12^ Sohn et al,^13^ and Hachiro et al^14^ found no significant differences in mortality, graft patency, or MACCE between ipsilateral and contralateral grafts. However, these studies were limited by either small sample sizes, non-US populations, or comparisons to solely contralateral grafts. Our study, representing the largest US-based analysis to date and accounting for free grafts, supports previous findings, reinforcing the view that ipsilateral in situ ITA grafts can be used safely in select patients, and that while coronary steal may occur, its clinical impact is likely limited.

Although Shim et al^11^ reported increased rates of haemodialysis-induced angina and cardiac deaths with ipsilateral ITA grafts in a Korean cohort, our study did not replicate these findings. This discrepancy may reflect differences in patient characteristics, dialysis protocols, or surgical practices, highlighting the importance of careful patient selection. Preoperative imaging should be performed whenever possible to evaluate for subclavian stenosis, as it may alter haemodynamics during dialysis and negatively impact outcomes. In our study, patients with subclavian stenosis who received ipsilateral grafts demonstrated higher rates of MACCE. However, due to limited statistical power, we were unable to formally assess the significance of this association. Additionally, AV fistula characteristics such as location (eg, brachiocephalic) and flow rate (>1.5-2.0 L/min) should also be considered, as these may increase the likelihood of coronary steal.^15^^,^^16^ Although our dataset lacked sufficient granularity to assess these factors, future studies using larger, more detailed datasets should investigate their influence on outcomes. Intraoperative flow measurements can help confirm adequate graft perfusion under baseline and stress conditions. Additionally, patients with impaired cardiac function may be more susceptible to ischaemic complications from altered ITA flow and warrant caution. Together, these measures support a tailored, risk-informed approach to graft selection in this complex patient population.

Patients with ESRD often have heavily calcified, atherosclerotic aortas, and one of the major advantages of using ipsilateral in situ ITAs in this patient population is avoiding the embolic risk associated with free graft anastomosis.^17^^,^^18^ Additionally, using a contralateral ITA in situ (typically the right ITA when the AV fistula is in the left, non-dominant arm) may not always be feasible due to graft length limitations. In this context, the observed equivalence in outcomes between ipsilateral in situ ITA grafts and alternative configurations is reassuring, as it may help avoid the risks associated with free grafting, and improve graft-choice flexibility in scenarios where contralateral ITA use may not be possible.

Our study is limited by a modest sample size, which may have reduced our ability to detect significant associations between ITA-AVF laterality and the outcomes of interest. The limited cohort and number of outcome events also restricted the number of covariates that could be included in our multivariable regression models, raising the likelihood of unmeasured confounders that could have influenced our results. Several outcome variables in our study also demonstrated between-group differences that ultimately did not reach statistical significance, likely due to limited power. Future larger studies will be needed to better evaluate these associations. Further, the contralateral and free ITA groups were combined to preserve statistical power and because both represent strategies that avoid ipsilateral in situ ITA dependence in the setting of an UE-AVF, but this grouping may have ignored residual heterogeneity within the cohort, as contralateral in situ and free ITA grafts differ with respect to inflow source, conduit handling, aortic manipulation, and patient selection. Additionally, approximately one-third of patients underwent concomitant valve or other cardiac procedures, which introduces additional clinical heterogeneity and may influence postoperative outcomes independent of ITA-AVF laterality. We attempted to ameliorate this by performing an additional unadjusted sensitivity analysis limited to patients undergoing isolated CABG. This analysis demonstrated a similar overall pattern, with no apparent excess in haemodialysis-induced ischaemia or 3-year mortality among patients receiving ipsilateral in situ ITA grafts. Although MACCE was lower in the ipsilateral group in this subgroup, this finding should be interpreted cautiously. The analysis was unadjusted, involved fewer patients and events, and remains susceptible to selection bias and residual confounding. Therefore, these subgroup findings should be viewed as supportive of the primary descriptive analysis rather than evidence of a protective effect of ipsilateral grafting. Finally, as a dual-centre study, our findings may reflect institutional practices that may not be fully generalizable to the broader US population. However, the study remains the largest known analysis of this question in a US cohort and provides valuable insight into a clinically relevant but understudied issue, thereby laying the groundwork for future multicentre investigations.

In this retrospective cohort, ipsilateral in situ ITA grafting was not associated with worse perioperative or long-term outcomes compared with contralateral in situ or free ITA grafting. However, because of the modest sample size and potential for residual confounding, these findings should be viewed as descriptive and hypothesis-generating rather than definitive evidence of equivalence. Ipsilateral in situ ITA grafting may be considered in carefully selected patients after individualized assessment of subclavian disease, AVF characteristics, conduit options, and intraoperative graft flow.

## Supplementary Material

ivag231_Supplementary_Data

## Data Availability

The data underlying this article are available in the article and in its online supplementary material.
