# Supplementary material for: Surgical Outcomes of in situ Internal Thoracic Artery Grafting in Patients with Ipsilateral Upper-Extremity Arteriovenous Fistulas
Source: Interdiscip Cardiovasc Thorac Surg. 2026 Aug 26;41(9):ivag231. doi: 10.1093/icvts/ivag231 (PMC13541775; doi:10.1093/icvts/ivag231)
Supplement: Supplementary file 1 [file ivag231_supplementary_data.zip › Supplementary Tables.docx]

**Supplementary Table 1: Univariable analysis for factors associated with MACCE**

|  | **OR (95% CI)** | **p value** |
| --- | --- | --- |
| Age | 1.00 (0.96, 1.03) | 0.813 |
| BMI | 0.99 (0.87, 1.13) | 0.927 |
| Female sex | 1.87 (0.74, 4.73) | 0.189 |
| Race |  |  |
| White | Ref |  |
| Black | 1.10 (0.28, 4.22) | 0.895 |
| Asian | 1.10 (0.17, 6.96) | **0.023** |
| Hispanic | 1.03 (0.31, 3.45) | 0.966 |
| White Race | 0.73 (0.32, 1.68) | 0.459 |
| Hypertension | 0.15 (0.02, 1.44) | 0.101 |
| Diabetes | 1.06 (0.41, 2.72) | 0.907 |
| Atrial Fibrillation | 0.99 (0.37, 2.67) | 0.985 |
| Creatinine | 0.97 (0.81, 1.17) | 0.751 |
| Peripheral vascular disease | 0.67 (0.28, 1.61) | 0.368 |
| Chronic lung disease | 0.30 (0.08, 1.13) | 0.076 |
| History of smoking | 0.60 (0.27, 1.30) | 0.195 |
| Congestive Heart Failure | 0.89 (0.41, 1.94) | 0.766 |
| LVEF | 0.98 (0.95, 1.02) | 0.309 |
| Stroke | 1.88 (0.59, 6.04) | 0.289 |
| Hyperlipidemia | 1.38 (0.33, 5.84) | 0.662 |
| Acute Coronary Syndrome | 1.49 (0.68, 3.28) | 0.323 |
| Prior PCI | 1.02 (0.45, 2.30) | 0.957 |
| Obstructive sleep apnea | 0.70 (0.30, 1.67) | 0.424 |
| Liver disease | 1.52 (0.29, 7.93) | 0.616 |
| Number of Diseased Vessels |  |  |
| 1 | Ref |  |
| 2 | 1.83 (0.38, 8.78) | 0.448 |
| 3 or more | 1.57 (0.38, 6.59) | 0.535 |
| Side of Arteriovenous Fistula |  |  |
| Right | 1.30 (0.58, 2.94) | 0.523 |
| Left | Ref |  |
| **Operative Characteristics** | | |
| Redo Sternotomy | 0.48 (0.05, 4.81) | 0.536 |
| Elective procedure | 0.96 (0.43, 2.14) | 0.911 |
| CPB time | 0.99 (0.99, 1.01) | 0.813 |
| Cross clamp time | 0.99 (0.99, 1.01) | 0.549 |
| Concomitant Operations |  |  |
| None | Ref |  |
| More than 1 | 1.01 (0.16, 6.43) | 0.99 |
| MV repair/replacement | 0.91 (0.20, 4.11) | 0.903 |
| AV repair/replacement | 1.21 (0.30, 4.90) | 0.786 |
| Other | 1.08 (0.31, 3.74) | 0.899 |
| SVG use |  |  |
| To OM | 1.14 (0.46, 2.83) | 0.777 |
| To Diag | 1.45 (0.55, 3.78) | 0.45 |
| All Arterial Conduits | 0.24 (0.05, 1.12) | 0.069 |

**Supplementary Table 2: Univariable analysis for factors associated with 3-year mortality**

|  | **HR (95% CI)** | **p value** |
| --- | --- | --- |
| Age | 0.99 (0.96, 1.03) | 0.841 |
| BMI | 0.99 (0.90, 1.11) | 0.949 |
| Female sex | 1.44 (0.66, 3.12) | 0.361 |
| Race |  |  |
| White | Ref |  |
| Black | 0.27 (0.04, 1.95) | 0.192 |
| Asian | 0.98 (0.13, 7.21) | 0.981 |
| Hispanic | 0.46 (0.11, 1.94) | 0.292 |
| White Race | 2.17 (0.83, 5.66) | 0.113 |
| Hypertension | 1.68 (0.23, 12.4) | 0.609 |
| Diabetes | 1.12 (0.46, 2.72) | 0.811 |
| Atrial Fibrillation | 1.01 (0.41, 2.46) | 0.984 |
| Creatinine | 0.95 (0.80, 1.14) | 0.585 |
| Peripheral vascular disease | 2.10 (1.03, 4.26) | **0.041** |
| Chronic lung disease | 1.61 (0.66, 3.94) | 0.294 |
| History of smoking | 1.22 (0.60, 2.47) | 0.589 |
| Congestive Heart Failure | 2.24 (1.00, 5.00) | 0.05 |
| LVEF | 0.94 (0.91, 0.97) | **<0.0001** |
| Stroke | 1.31 (0.51, 3.42) | 0.576 |
| Hyperlipidemia | 0.74 (0.26, 2.13) | 0.582 |
| Acute Coronary Syndrome | 0.75 (0.37, 1.52) | 0.43 |
| Prior PCI | 0.80 (0.38, 1.70) | 0.562 |
| Obstructive sleep apnea | 1.09 (0.51 2.32) | 0.822 |
| Liver disease | 1.60 (0.49, 5.27) | 0.439 |
| Number of Diseased Vessels |  |  |
| 1 | Ref |  |
| 2 | 1.87 (0.40, 8.66) | 0.423 |
| 3 or more | 1.32 (0.31, 5.65) | 0.708 |
| Side of Arteriovenous Fistula |  |  |
| Right | 0.64 (0.28, 1.42) | 0.27 |
| Left | Ref |  |
| **Operative Characteristics** | | |
| Redo Sternotomy | 1.13 (0.15, 8.29) | 0.905 |
| Elective procedure | 0.75 (0.34, 1.62) | 0.459 |
| CPB time | 0.99 (0.99, 1.00) | 0.312 |
| Cross clamp time | 0.99 (0.99, 1.01) | 0.385 |
| Concomitant Operations |  |  |
| None | Ref |  |
| More than 1 | 1.06 (0.14, 7.87) | 0.958 |
| MV repair/replacement | 1.57 (0.47, 5.29) | 0.466 |
| AV repair/replacement | 1.80 (0.68, 4.79) | 0.241 |
| Other | 0.71 (0.17, 3.02) | 0.64 |
| LIMA use LAD vs others | 1.02 (0.14, 7.49) | 0.985 |
| RIMA use yes vs no | 2.28 (0.80, 6.54) | 0.124 |
| SVG use |  |  |
| To OM | 0.43 (0.20, 0.92) | **0.029** |
| To Diag | 1.09 (0.46, 2.59) | 0.839 |
| Bilateral ITA use | 1.79 (0.55, 5.91) | 0.336 |
| All Arterial Conduits | 1.10 (0.38, 3.15) | 0.859 |

**Supplementary Table 3: Multivariable logistic regression assessing association between AV-fistula laterality and incidence of MACCE**

|  | **Univariable** | | **Multivariable** | |
| --- | --- | --- | --- | --- |
|  | **OR (95% CI)** | **P-value** | **aOR (95% CI)** | **P-value** |
| Ipsilateral AV fistula | 0.54 (0.25, 1.18) | 0.124 | 0.68 (0.32, 1.44) | 0.31 |
| Age | 1.00 (0.96, 1.03) | 0.813 | 1.01 (0.97, 1.05) | 0.695 |
| Diabetes | 1.06 (0.41, 2.72) | 0.907 | 1.14 (0.45, 2.87) | 0.778 |
| Hypertension | 0.15 (0.02, 1.44) | 0.101 | 0.18 (0.02, 1.77) | 0.141 |
| Smoking History | 0.60 (0.27, 1.30) | 0.195 | 0.68 (0.32, 1.44) | 0.31 |
| Peripheral Vascular Disease | 0.67 (0.28, 1.61) | 0.368 | 0.96 (0.43, 2.12) | 0.907 |
| Hyperlipidemia | 1.38 (0.33, 5.84) | 0.662 | 1.19 (0.34, 4.15) | 0.784 |

**Supplementary Table 4: Multivariable logistic regression assessing association between AV-fistula laterality and 3-year mortality**

|  | **Univariable** | | **Multivariable** | |
| --- | --- | --- | --- | --- |
|  | **HR (95% CI)** | **P-value** | **aHR (95% CI)** | **P-value** |
| Ipsilateral AV fistula | 1.49 (0.72, 3.07) | 0.281 | 1.56 (0.68, 3.58) | 0.291 |
| Peripheral Vascular Disease | 2.10 (1.03, 4.26) | **0.041** | 1.61 (0.71, 3.65) | 0.259 |
| LVEF | 0.94 (0.91, 0.97) | **<0.0001** | 0.94 (0.90, 0.97) | **0.0001** |

**Supplementary Table 5: Unadjusted outcomes for patients who underwent isolated CABG**

|  | **Overall (n= 82)** | **Ipsilateral (n=47)** | **Contralateral + Free ITA (n= 37)** | **p value** |
| --- | --- | --- | --- | --- |
| HD induced ischemia | 2 (2%) | 1 (2%0 | 1 (3%) | 1 |
| MACE | 36 (44%) | 16 (34%) | 20 (57%) | **0.037** |
| 3 year mortality | 20 (24%) | 11 (23%) | 9 (26%) | 0.81 |
